# Structural relationships between emotional intelligence, well-being, and psychological distress: a multi-group SEM study among undergraduate physical education students in three Arab countries

**DOI:** 10.3389/fpsyg.2025.1650030

**Published:** 2025-10-03

**Authors:** Kashef Zayed, Ehab Omara, Mahfoodha Al Kitani, Ali Al-Yaaribi, Khalifa Al-Jadidi, Abdul Rahim Daroushi, Majid Al Busafi, Amin Gaafar, Heba El-Ashkar, Badriya Al-Hadabi, Ezzedin Ali

**Affiliations:** ^1^Department of Physical Education and Sport Sciences, College of Education, Sultan Qaboos University, Muscat, Oman; ^2^Department of Psychology, College of Education, Sultan Qaboos University, Muscat, Oman; ^3^Humanities Research Center, Sultan Qaboos University, Muscat, Oman

**Keywords:** emotional intelligence, psychological well-being, psychological distress, cross-cultural, structural equation modeling, university students

## Abstract

**Background:**

Emotional intelligence (EI) has been consistently associated with higher levels of psychological well-being (WB) and lower levels of psychological distress (PD). Yet, relatively little is known about how these relationships operate within Arab cultural contexts, especially among students training to become professionals in fields that promote both mental and physical health. Given the growing recognition of mental health challenges among youth in the region, examining these associations within Arab university settings is both timely and important. This study aimed to investigate how EI, WB, and PD are connected in undergraduate physical education students from Oman, Kuwait, and Jordan. It also examined whether WB helps explain the link between EI and PD and whether the proposed model holds consistently across these different cultural settings.

**Methods:**

A cross-sectional survey design was used with a sample of 788 undergraduate physical education students. Participants completed validated self-report measures assessing EI (five theoretically grounded dimensions: Self-Awareness, Empathy, Self-Regulation, Social Regulation, and Emotional Control), psychological well-being (Short Warwick-Edinburgh Mental Well-Being Scale), and psychological distress (Depression, Anxiety, and Stress Scale). Confirmatory Factor Analysis (CFA) evaluated the measurement models, and Structural Equation Modeling (SEM) tested direct and indirect relationships. Multi-Group Confirmatory Factor Analysis (MGCFA) examined measurement invariance across the three countries. To provide a more comprehensive picture of the associations, Pearson correlations and multiple regression analyses were also conducted.

**Results:**

CFA confirmed the validity and reliability of the measures across all three samples. The SEM results indicated that EI was directly and positively related to WB, and indirectly related to PD through the mediating effect of WB. While configural and metric invariance were established, scalar invariance was not, limiting mean-level comparisons across countries. Nonetheless, the achieved invariance allowed valid structural analyses. Mediation analysis revealed that WB significantly mediated the EI– PD relationship in Kuwait, but not in Oman or Jordan. These findings suggest that the strength and direction of psychological mechanisms may vary by cultural context.

**Conclusion:**

The study highlights the positive role of EI in enhancing well-being and reducing psychological distress among university students in Arab contexts. However, the pathways linking these constructs may differ across cultural settings. Practical implications include the integration of emotional intelligence development into university curricula, particularly in disciplines that emphasize both mental and physical health. These findings emphasize the need for culturally sensitive approaches to emotional intelligence training and mental health promotion, especially for students preparing to serve as future educators and health professionals.

## Introduction

University life is a key stage in both personal growth and academic development. It offers students valuable opportunities to expand their knowledge, build social connections, and shape their sense of identity. At the same time, this period often brings increased exposure to psychological challenges. Factors such as academic pressure, adjusting to new social environments, and the shift toward greater independence can contribute to higher levels of stress, anxiety, and depression. These mental health concerns are not limited to the early years of university; in many cases, they tend to intensify as students progress through their academic life (Ibrahim et al., 2013; Ünlü Kaynakçı and Yerin Güneri, 2023). In Arab countries like Oman, Kuwait, and Jordan, students may also experience additional stressors related to sociocultural expectations, stigma surrounding mental health issues, and limited access to professional psychological support (Okasha et al., 2012).

In response to these challenges, emotional intelligence (EI) has been seen as a possible protective factor for mental health. EI refers to a person’s ability to recognize, understand, manage, and use emotions effectively in themselves and in their relationships. Emotional intelligence is commonly conceptualized in two main ways: as an ability, focusing on the cognitive processing of emotional information, and as a trait, emphasizing self-perceived emotional competencies (Mayer et al., 2004; Petrides et al., 2016). In the present study we adopted the trait perspective, using self-report instruments to capture individuals’ perceptions and everyday application of their emotional skills.

A growing body of literature has shown that people with higher EI report better psychological wellbeing, more effective coping strategies, and lower levels of distress (Malinauskas and Malinauskiene, 2020; Caballero-García and Ruiz, 2025; Shrestha, 2025). In university settings, students with high emotional intelligence tend to navigate academic and social demands more successfully, experiencing lower stress and higher life satisfaction (Bereded et al., 2025; Akyıl and İme, 2024; Shahin, 2020). These benefits may be particularly important for students in health-related fields, as they often deal with both academic demands and practical training.

This study focuses on undergraduate students studying physical education in Oman, Kuwait, and Jordan. This group was selected for several reasons. First, physical education students are training to become future educators and promoters of both mental and physical health; their own psychological well-being is therefore professionally relevant. Second, these students often face unique stress due to the combined demands of academic study and physical performance. Third, their programs often include psychological and emotional skill-building, such as motivation, resilience, and empathy, which are closely related to emotional intelligence and may be influenced by local cultural values and teaching approaches (Ciotto and Gagnon, 2018). Physical education helps students build teamwork, empathy, resilience, and motivation, which are core components of emotional intelligence as highlighted by Ciotto and Gagnon (2018) and Rico-González (2023). This makes physical education a highly relevant context for examining how emotional intelligence influences psychological functioning in academic and professional life. Studying emotional intelligence, well-being, and psychological distress in this specific group provides culturally meaningful insight into how emotional competencies support mental health in a region that has not yet been well studied in this area. Evidence suggests that EI functions as a protective factor against stress, anxiety, and depression in higher education settings (Malinauskas and Malinauskiene, 2020; Ünlü Kaynakçı and Yerin Güneri, 2023). Within physical education, EI has further been associated with fostering resilience, enhancing motivation, and supporting social–emotional learning (Ciotto and Gagnon, 2018; Rico-González, 2023).

Therefore, the aim of this study is to investigate the direct and indirect relationships between emotional intelligence, psychological well-being, and psychological distress in physical education undergraduates across three Arab countries. The study also seeks to assess the cross-cultural validity and measurement equivalence of these variables using SEM and MGCFA. These findings can help us better understand how emotions work in learning environments that value social connection, and guide mental health interventions for future physical education professionals in the Arab world (Ryan and Deci, 2001).

## Hypotheses

According to Self-Determination Theory (Deci and Ryan, 2000) emotional intelligence functions as a fundamental factor which elevates psychological well-being through the satisfaction of basic psychological needs including autonomy competence and relatedness. Multiple research studies have shown that people who experience greater well-being display reduced psychological distress (Keyes, 2005). According to mediation studies psychological well-being seems to function as a mediator that links emotional intelligence to psychological distress (Zayed et al., 2023). Our research proposes three hypotheses based on theoretical background and supporting evidence:

*H1*: Emotional intelligence shows a direct positive association with psychological well-being.

*H2*: People with greater psychological well-being experience lower psychological distress.

*H3*: Psychological well-being functions as a mediator in the relationship between emotional intelligence and psychological distress.

### Objectives

This study had three primary objectives: (1) to assess the factorial validity and internal reliability of the instruments used to measure EI, WB, and PD through Confirmatory Factor Analysis (CFA); (2) to examine the direct and indirect relationships among EI, WB, and PD using Structural Equation Modeling (SEM), with particular attention to the mediating role of WB in the association between EI and PD; and (3) to evaluate the cross-cultural measurement and structural invariance of the proposed model across the three national samples using Multi-Group Confirmatory Factor Analysis (MGCFA).

By addressing these objectives, the study contributes to the expanding literature on emotional functioning and student mental health in non-Western cultural settings. The findings provide empirical support for the use of established psychological constructs within Arab higher education contexts and offer insights that can inform the design of culturally responsive EI interventions aimed at promoting mental well-being among university students.

## Materials and methods

### Participants

**Figure 1:**
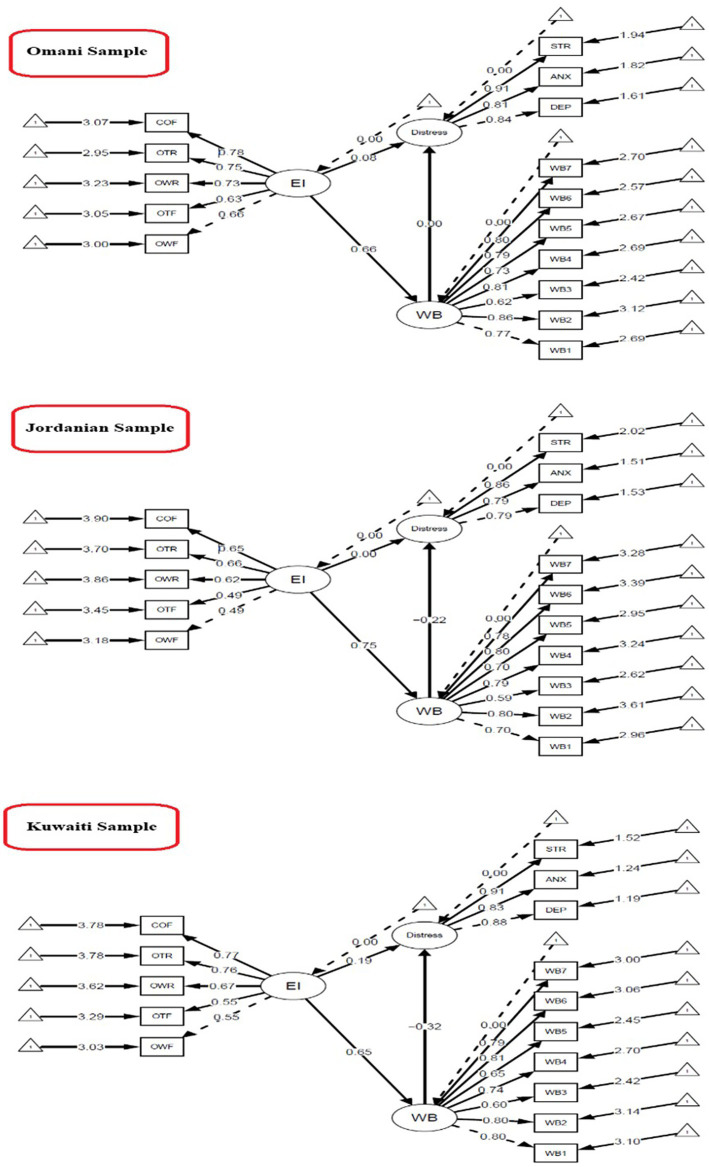
Mediation model of emotional intelligence, psychological distress, and well-being across Omani, Jordanian, and Kuwaiti samples.

A total of 788 undergraduate students majoring in physical education participated in this study. Data collection took place between March and April 2024. Invitations distributed through faculty email lists at selected universities in Oman, Kuwait, and Jordan, and the overall response rate was approximately 78% of the invited students. Upon accessing the online survey link, participants were presented with an informed consent form describing the purpose of the study, their rights as participants, and assurances of confidentiality. Only those who agreed and signed the informed consent were able to proceed. The survey first gathered demographic information, followed by standardized instruments assessing emotional intelligence, psychological well-being, and psychological distress. Eligibility was limited to undergraduate students who were currently enrolled in physical education programs and preparing to become physical education teachers in the future. The final sample consisted of 343 male and 445 female students. By country, 198 students were from Oman, 204 from Jordan, and 386 from Kuwait. Representation was achieved across all academic years. The mean age of participants was 21.3 years (SD = 1.9) (Figure 1).

### Measures

#### Emotional intelligence

Emotional intelligence was assessed using the Brief Emotional Intelligence Scale (BEIS-10), a 10-item self-report instrument developed by Davies et al. (2010) as a short-form version of the original 33-item Emotional Intelligence Scale by Schutte et al. (1998). The BEIS-10 measures five core dimensions of emotional intelligence, each represented by two items, and provides an overall EI score. The dimensions are:

- Appraisal of Own Emotions (e.g., “I easily recognize my emotions as I experience them”).
- Appraisal of Others’ Emotions (e.g., “By looking at their facial expressions, I recognize the emotions people are experiencing”).
- Regulation of Own Emotions (e.g., “I seek out activities that make me happy”).
- Regulation of Others’ Emotions (e.g., “I help other people feel better when they are down”).
- Utilization of Emotions (e.g., “When I am in a positive mood, I am able to come up with new ideas”).

Each item was rated on a 5-point Likert scale ranging from 1 (strongly disagree) to 5 (strongly agree). Higher scores indicate greater self-perceived emotional intelligence.

The BEIS-10 has demonstrated solid psychometric properties. The BEIS-10 was selected due to its implementation of a trait-based model of emotional intelligence, providing a concise and thorough evaluation appropriate for a broad administration. The instrument exhibits strong psychometric properties, demonstrating acceptable reliability and validity in both Western and Arab populations. The precise nature reduces the burden on respondents while ensuring sufficient coverage of the essential aspects of emotional intelligence. Confirmatory factor analysis in the original development study supported its five-factor structure (CFI = 0.97, NNFI = 0.94, RMSEA = 0.06), and internal consistency was acceptable (Cronbach’s *α* ranging from 0.64 to 0.79 for the subscales, and 0.75 for the overall scale). Test–retest reliability over a two-week interval also indicated temporal stability (intraclass correlation coefficient, ICC = 0.612; Davies et al., 2010).

In the present study, the Arabic version of the BEIS-10 was used. The scale was translated using a standard back-translation procedure to ensure linguistic and conceptual equivalence with the original.

English version. For our sample, Cronbach’s α values ranged from 0.64 to 0.72 across the five subscale scores, and 0.79 for the total EI score. A test–retest assessment on a subset of participants (over a 16 day interval) yielded an ICC of 0.663 for the total score, indicating acceptable measurement stability over time.

#### Psychological well-being

Psychological well-being was assessed using the Short Warwick-Edinburgh Mental Well-Being Scale (SWEMWBS). This is a 7-item self-report questionnaire developed to measure positive aspects of mental health in the general population. It focuses on experiences such as optimistic thinking, feeling useful, relaxation, dealing with problems, clear thinking, closeness to others, and confidence. The SWEMWBS is a shortened form of the original 14-item Warwick-Edinburgh Mental Well-Being Scale (WEMWBS), and has been shown to be both valid and reliable in diverse population (Vaingankar et al., 2017; Fung et al., 2025; Hanzlová and Lynn, 2023).

Participants were asked to rate how often they had experienced each of the seven statements over the past 2 weeks, using a 5-point Likert scale (1 = none of the time, 5 = all of the time). Higher scores indicate better mental well-being. Previous studies have reported that the SWEMWBS has good internal consistency (Cronbach’s *α* between 0.84 and 0.87) and a one-factor structure that holds across different populations.

In this study, we utilized the validated Arabic version of the SWEMWBS. This version was previously tested by Zayed et al. (2023), who found that it had good reliability and supported the same one-factor structure in a sample of undergraduate students from Oman, Qatar, and Saudi Arabia. These findings suggest that the Arabic SWEMWBS is suitable for use with university students in Arab countries. In our sample, the SWEMWBS total score demonstrated excellent reliability (Cronbach’s α = 0.90).

#### Psychological distress

Psychological distress was measured using the Depression, Anxiety, and Stress Scale–21 (DASS-21), a self-report instrument developed by Lovibond and Lovibond (1995) to assess three related negative emotional states: depression, anxiety, and stress. The DASS-21 is the short form of the original 42item DASS and is widely used in both clinical and non-clinical settings. It consists of three subscales, each comprising 7 items, corresponding to:

- Depression (e.g., “I felt that I had nothing to look forward to”).
- Anxiety (e.g., “I felt scared without any clear reason”).
- Stress (e.g., “I found it difficult to relax”).

Participants were required to state the degree to which each point was relevant to their experiences during the past week. A four-point Likert scale was used, where 0 represented ‘not applied at all’ and 3 - ‘applied very much or most of the time’. Consistent with the DASS-21 scoring recommendations, the items were allocated to the three subscales of depression, anxiety, and stress. The sum of scores for each subscale was calculated and then each score was converted to be comparable with the original 42item version by multiplying the result by two. A measure of overall psychological distress was derived from the sum of the three subscale scores. Higher scores both on the total scale and on the individual subscales indicate more severe psychological distress.

The DASS-21 has demonstrated robust psychometric properties across diverse populations. Its construct validity is supported by confirmatory factor analyses, and internal consistency is high for all subscales and the total score (Cronbach’s *α* typically ranging from 0.81–0.94 for Depression, 0.70–0.89 for Anxiety, 0.80–0.91 for Stress, and 0.87–0.95 for the total scale) (Henry and Crawford, 2005;Moussa et al., 2017). Short-term test–retest reliability studies have also confirmed the scale’s stability over time.

We used the Arabic version of the DASS-21 in this research. This version was developed through a rigorous translation and back-translation process to ensure conceptual and linguistic equivalence with the English original. In our sample, the Arabic DASS-21 showed acceptable internal consistency, with.

Cronbach’s α values of 0.68 (Depression), 0.74 (Anxiety), 0.76 (Stress), and 0.83 for the overall 21item scale.

### Procedure

This study received ethical approval from the Scientific Research Committee of the Department of Physical Education and Sports Sciences, College of Education, which functioned as the Institutional Review Board (Approval No: PHED/SRC/1862/05/2023). Undergraduate students from universities in Oman, Kuwait, and Jordan were electronically invited to participate. Prior to completing the survey, all participants were provided with a clear and comprehensive explanation of the study’s purpose, procedures, and measures taken to ensure confidentiality and data protection. Those who consented to participate did so by signing an electronic informed consent form. Only participants who provided consent were allowed to proceed to the self-report questionnaire, which assessed emotional intelligence, psychological well-being, and psychological distress. The survey required approximately 15–20 min to complete.

### Data analysis

All data were analyzed using R statistical software (Version 4.3.2). We employed several specialized packages, including lavaan, semTools, semPlot, and psych, for the SEM and psychometric analyses.

The analysis began with descriptive statistics and internal consistency assessments for each scale.

Cronbach’s *α* was computed for each measure and subscale, with a threshold of 0.70 considered indicative of acceptable reliability (George and Mallery, 2016). Skewness and kurtosis values were examined to evaluate univariate normality.

Next, a Confirmatory Factor Analysis (CFA) was conducted to evaluate the measurement model. We specified three latent constructs: Emotional Intelligence (five indicators corresponding to the BEIS-10 composite dimensions), Psychological Well-Being (seven indicators corresponding to the SWEMWBS items), and Psychological Distress (three indicators corresponding to the DASS-21 subscale composites for Depression, Anxiety, and Stress). The CFA used maximum likelihood estimation with robust (Huber-White) standard errors to account for any non-normality. Model fit was assessed using multiple indices: Comparative Fit Index (CFI) and Tucker-Lewis Index (TLI) with values ≥ 0.90 indicating acceptable fit (Hu and Bentler, 1999), Root Mean Square Error of Approximation (RMSEA) with values ≤ 0.08 (and its 90% confidence interval) indicating reasonable error of approximation, and Standardized Root Mean Square Residual (SRMR) with values ≤ 0.08 indicating good fit (Kline, 2023).

After confirming an adequate measurement model, we specified a structural equation model to examine the hypothesized structural relationships among the latent variables. In this structural model, Emotional Intelligence was modeled as a predictor of both Psychological Well-Being and Psychological Distress, and Psychological Well-Being was modeled as a predictor of Psychological Distress. In other words, we tested whether EI directly influences WB and PD, as well as whether EI influences PD indirectly through WB (mediation). Direct, indirect, and total effects were estimated. The significance of the indirect (mediated) effect was assessed using a non-parametric bootstrap approach with 1,000 resamples to generate bias-corrected confidence intervals (Preacher and Hayes, 2008).

Additionally, we performed Multi-Group Confirmatory Factor Analysis (MGCFA) to test measurement invariance of the constructs across the three countries (Oman, Kuwait, and Jordan). We tested for configural invariance (same pattern of factor loadings across groups), metric invariance (factor loadings constrained equal across groups), and scalar invariance (item intercepts constrained equal across groups) in a sequential manner. Model comparisons used changes in CFI (ΔCFI) and RMSEA (ΔRMSEA) to evaluate whether adding constraints significantly worsened model fit. We adopted the criteria ΔCFI ≤ 0.01 and ΔRMSEA < 0.015 as indicative of no substantial decrement in fit (Cheung and Rensvold, 2002) when moving from a less constrained to a more constrained model. If full scalar invariance was not achieved, we planned to examine modification indices and free a minimal set of intercepts to establish partial invariance, if theoretically justifiable.

Upon establishing at least metric invariance, we proceeded with multi-group SEM analyses to compare structural pathways across countries. We first fit a multi-group SEM allowing the structural coefficients to vary freely between groups, and then compared it to models with specific paths constrained equal across the three groups. Likelihood-ratio tests and changes in fit indices (especially ΔCFI and ΔRMSEA as noted above) were used to determine whether constraining a given path significantly degraded the model fit, which would suggest a cross-country difference in that relationship.

All significance tests were two-tailed, with a significance level of *p* < 0.05. Effect sizes for direct effects were interpreted with standardized coefficients (*β*), and mediation effect sizes were interpreted via standardized indirect effects. Where relevant, we report confidence intervals and *p*-values for indirect effects from the bootstrap analysis.

## Results

### Descriptive statistics, reliability, and construct validity

Descriptive statistics for the study variables are presented in Table 1. The sample comprised 788 participants from three countries (Oman, Kuwait, Jordan). The Mental Well-Being Scale.

**Table 1:** Descriptive statistics and reliability coefficients for study variables.

Variable	*M*	*SD*	Skewness	Kurtosis
Well-Being (WB) - (*α* = 0.90)				
WB1	3.75	1.28	−0.70	−0.65
WB2	3.73	1.16	−0.63	−0.46
WB3	2.94	1.20	0.13	−0.85
WB4	3.34	1.19	−0.24	−0.84
WB5	3.19	1.23	−0.11	−0.93
WB6	3.64	1.23	−0.59	−0.67
WB7	3.7	1.26	−0.71	−0.57
Well-Being (WB) - (*α* = 0.90)				
OWF	3.34	1.09	−0.23	−0.84
OTF	3.5	1.07	−0.37	−0.68
OWR	3.46	0.97	−0.52	−0.21
OTR	3.5	0.99	−0.52	−0.33
COF	3.7	1.03	−0.84	0.08
Psychological Distress (PD) - (*α* = 0.89)				
DEP	0.99	0.73	0.67	−0.21
ANX	1.07	0.76	0.58	−0.3
STR	1.25	0.73	0.31	−0.57

(SWEMWBS-7) demonstrated excellent internal consistency (*α* = 0.899), with item means ranging from *M* = 2.94 (SD = 1.20) for WB3 (“I felt calm”) to *M* = 3.75 (SD = 1.28) for WB1 (“I felt optimistic about the future”). The Psychological Distress Scale also showed strong reliability (*α* = 0.891), with stress (*M* = 1.25, SD = 0.73) being the most frequently endorsed subscale. Emotional Intelligence (*α* = 0.785) exhibited acceptable reliability, with the highest mean for COF (“Control of Feelings”; *M* = 3.70, SD = 1.03). Skewness and kurtosis values for all variables fell within acceptable ranges (|skewness| < 1.0; |kurtosis| < 1.0), indicating no severe deviations from normality (Curran et al., 1996).

In addition to Cronbach’s alpha, composite reliability (CR) and average variance extracted (AVE) were computed to further assess construct reliability and convergent validity (Cheung et al., 2024). As shown in Table 2, CR values exceeded the recommended threshold of 0.70 across all constructs, ranging from 0.79 (Emotional Intelligence) to 0.90 (Well-being), supporting adequate reliability. AVE values indicated satisfactory convergent validity for Well-being (0.566) and Psychological Distress (0.735), while Emotional Intelligence showed a marginally lower AVE (0.437), suggesting limited convergence among its indicators.

**Table 2:** Reliability, convergent validity, and discriminant validity (Fornell-Larcker criterion).

Construct	α	CR	AVE	EI	WB	PD
EI	0.785	0.793	0.437	**0.661**	–	–
WB	0.899	0.900	0.566	0.668	**0.752**	–
PD	0.891	0.893	0.735	−0.032	−0.159	**0.857**

α = Cronbach’s alpha; CR = Composite Reliability; AVE = Average Variance Extracted. Bold diagonal values are √AVE; off-diagonals are latent construct correlations.

Discriminant validity was evaluated using the Fornell–Larcker criterion (Fornell and Larcker, 1981; see Table 2). The square root of AVE for each construct (diagonal values) exceeded its correlations with other constructs, except for Emotional Intelligence, where √AVE (0.661) was slightly lower than its correlation with Well-being (0.668). This indicates generally acceptable discriminant validity, although the overlap between Emotional Intelligence and Well-being warrants cautious interpretation.

### Confirmatory factor analysis for the validation of measurement models

To validate the internal structure of the developed instruments, a single confirmatory factor analysis.

(CFA) model was specified, comprising three correlated latent variables: Emotional Intelligence (EI), Well-Being (WB), and Psychological Distress (PD). Each construct was measured by a distinct set of items or components, and the model was evaluated to confirm the adequacy of the proposed factor structure before proceeding to examine the interrelations among these constructs.

The model demonstrated an acceptable fit to the data: *χ*^2^(87) = 433.70, *p* < 0.001; CFI = 0.942; TLI = 0.930; RMSEA = 0.071 [90% CI: 0.065–0.078]; SRMR = 0.048, consistent with commonly recommended criteria (Hu and Bentler, 1999). All standardized factor loadings (*λ*) were statistically significant (*p* < 0.001) and exceeded the recommended minimum value of 0.50 (Hair et al., 2019), providing strong evidence for convergent validity across the three constructs. Factor loadings ranged from 0.555 to 0.742 for EI, 0.604 to 0.816 for WB, and 0.819 to 0.896 for PD.

The pattern of covariances among the latent variables indicated that EI was positively associated with WB (*r* = 0.668, *p* < 0.001), while WB was inversely associated with PD (*r* = −0.159, *p* < 0.001). The nonsignificant direct association between EI and PD (*r* = −0.032, *p* = 0.458) suggests a possible mediating role for WB in the relationship between EI and psychological distress. Full results, including standardized and unstandardized loadings, standard errors, and significance levels, are presented in Table 3.

**Table 3:** Confirmatory factor analysis results: model fit indices and standardized factor loadings.

Latent variables	Item	Std. Loading (*λ*)	Unstd. Loading (β)	SE	*p*
Emotional intelligence (EI)					
	OWF	0.555	1	—	—
	OTF	0.564	0.999	0.085	< 0.001
	OWR	0.680	1.089	0.08	< 0.001
	OTR	0.739	1.215	0.088	< 0.001
	COF	0.742	1.262	0.09	< 0.001
Well-Being (WB)					
	WB1	0.773	1	—	—
	WB2	0.816	0.954	0.039	< 0.001
	WB3	0.604	0.732	0.043	< 0.001
	WB4	0.770	0.929	0.042	< 0.001
	WB5	0.677	0.841	0.044	< 0.001
	WB6	0.801	0.999	0.042	< 0.001
	WB7	0.799	1.015	0.043	< 0.001
Psychological distress (PD)					
	DEP	0.856	1	—	—
	ANX	0.819	0.998	0.037	< 0.001
	STR	0.896	1.043	0.036	< 0.001
Model fit indices	χ^2^ (df) = 433.70 (87)**, CFI = 0.942, TLI = 0.930, RMSEA [90% CI] = 0.071 [0.065–0.078], SRMR = 0.048				

β = Standardized loading, λ = Unstandardized loading. ***p < 0.001, **p < 0.01, *p < 0.05.

### Multi-group invariance testing

The multi-group invariance analysis was conducted following a hierarchical sequence to assess the cross-cultural equivalence of the measurement and structural models. Initially, the results supported configural invariance, as indicated by acceptable model fit indices (*χ*^2^ (261) = 699.76, CFI = 0.942, RMSEA = 0.071), confirming that the hypothesized three-factor: structure Emotional Intelligence (EI), Well-Being (WB), and Psychological Distress (Distress)—was consistently represented across samples from Oman, Kuwait, and Jordan. Subsequently, testing for metric invariance, which imposes equality constraints on factor loadings, revealed no significant decline in model fit (Δχ^2^ (24) = 27.09, *p* = 0.301; RMSEA = 0.072, CFI = 0.940). This finding supports the equivalence of the structural relationships (e.g., regression paths and covariances) across the three cultural groups, thereby justifying crossnational comparisons of mediation effects (e.g., the indirect pathway from EI to Distress through WB). However, the test for scalar invariance, which constrains item intercepts to be equal across groups, was not supported (Δχ^2^ (24) = 78.64, *p* < 0.001; RMSEA = 0.093, CFI = 0.912), suggesting potential cultural biases in item interpretation that may confound comparisons of latent means (e.g., mean levels of EI or WB). Despite the lack of scalar invariance, the establishment of metric invariance allows for meaningful investigation of mediation models across the studied countries, as the structural parameters governing these pathways were statistically equivalent. The detailed fit indices and chi-square difference tests for each level of invariance are summarized in Table 4.

**Table 4:** Multi-group invariance testing across countries (Oman, Kuwait, Jordan).

Model	χ^2^ (*df*)	Δχ^2^ (*Δdf*)	*p*	RMSEA	CFI
Configural invariance	699.76 (261)	—	—	0.071	0.942
Metric invariance	726.84 (285)	27.09 (24)	0.301	0.072	0.94
Scalar invariance	805.48 (309)	78.64 (24)	< 0.001	0.093	0.912

### Cross-country mediation comparison

The multi-group mediation analysis revealed significant cross-country differences in the mechanisms linking emotional intelligence (EI), mental well-being (WB), and psychological distress, Δχ^2^ (6) = 13.86, *p* = 0.031. As shown in Table 5, the standardized indirect effects (EI → WB → Distress) varied substantially across Oman, Jordan, and Kuwait, suggesting cultural moderation of the mediation pathways.

**Table 5:** Multi-group mediation analysis results.

Country	*a* (EI → WB)	*b* (WB → Distress)	Indirect Effect (*a* × *b*)	Direct Effect (EI → Distress)
Oman	0.657***	0.001	0.0004	0.076
Jordan	0.746***	−0.217	−0.162	0.004
Kuwait	0.651***	−0.318**	−0.207**	0.189*

Path labels: EI = Emotional Intelligence, WB = Mental Well-Being. ***p < 0.001, **p < 0.01, *p < 0.05. Bootstrapped standard errors (1,000 resamples).

The multi-group analysis revealed distinct mediation patterns across countries. In Oman, the indirect effect of emotional intelligence (EI) on psychological distress through mental well-being (WB) was statistically non-significant (*β* = 0.0004, *p* = 0.996), indicating no mediation. In Kuwait, the indirect effect was significant (β = −0.207, *p* < 0.001), suggesting partial mediation (i.e., WB partially explained the EI-distress relationship), alongside a persistent direct effect (*β* = 0.189, *p* = 0.040). For Jordan, the indirect effect did not reach statistical significance (*β* = −0.162, *p* = 0.062), and the direct effect was negligible (*β* = 0.004, *p* = 0.983). Methodological factors, such as smaller sample sizes in Oman and Jordan compared to Kuwait, may have limited statistical power to detect mediation effects, particularly in Jordan, where the *p*-value approached the conventional threshold. These findings highlight the need for replication in larger, equivalently powered samples to clarify cross-national differences and reduce Type II error risks (Cohen, 1992; Fritz and MacKinnon, 2007). Future studies should prioritize robust sample sizes to enhance the reliability of mediation analyses in culturally diverse contexts.

## Discussion

This study examined the structural relationships between Emotional Intelligence (EI), Psychological Well-Being (WB), and Psychological Distress (PD) among undergraduate students in Oman, Kuwait, and Jordan. The findings support the growing body of evidence that emotional competencies are important contributors to mental health across diverse cultural contexts, particularly among Arab university students.

In the current research, it has been noted that students with high emotional intelligence (EI) not only reported better psychological well-being (WB) but also their EI was associated with lower psychological distress (PD), all this is in line with theoretical models and previous studies. Nevertheless, this intermediating relationship was found only in the Kuwaiti sample. These results are to some extent compatible with the findings of Akyıl and İme (2024) who pointed out the crucial role of well-being as a mediator in the EI–PD relationship. On the whole, the results indicate that the practice of recognizing, regulating, and employing feelings may, under particular circumstances, lead to increased well-being and thereby indirectly relieve the psychological distress. Still, this inclination should not be seen as generalized and its occurrence might be different across cultures.

Measurement models for EI, WB, and PD demonstrated strong construct validity and internal consistency across the three national samples, as confirmed by Confirmatory Factor Analysis (CFA). All factor loadings met recommended thresholds, indicating that these psychological constructs are conceptualized similarly across Arab cultures. These findings contribute to growing cross-cultural validation research for EI and well-being instruments (Malinauskas and Malinauskiene, 2020).

The structural model further confirmed a robust positive association between EI and WB across all countries (Ryan and Deci, 2001). This aligns with prior research suggesting that emotionally intelligent individuals are better equipped to manage stress, sustain positive affect, and adopt health-promoting behaviors—all of which enhance mental well-being (Ünlü Kaynakçı and Yerin Güneri, 2023). In Arab cultures, where emotional restraint and social cohesion are culturally valued (Matsumoto, 2006; Okasha et al., 2012), emotional competencies may be especially important for navigating interpersonal and internal experiences.

Regionally, our findings align with emerging studies confirming the relevance of EI among Arab youth. For instance, Nassar et al. (2024) demonstrated the validity of EI among Middle Eastern young adults, while Shahin (2020) reported a negative correlation between EI and perceived stress among Saudi university students. Additionally, recent research during the Gaza war has documented elevated distress among university students in Jordan and Egypt, reinforcing the importance of emotional resilience during periods of sociopolitical turmoil (Hendawy et al., 2024; Tanashat et al., 2024).

The mediating role of WB provides a more nuanced understanding of how EI contributes to mental health. From the perspective of Self-Determination Theory (Deci and Ryan, 2000), emotional skills may enhance well-being by supporting basic psychological needs—such as autonomy, competence, and relatedness—which in turn reduce psychological distress. Building on this perspective, the educational setting—and particularly physical education—represents a key context in which emotional and social competencies can be refined. Through social interaction, cooperation, and the regulation of emotions within performance contexts, physical education provides opportunities for students to strengthen EI, with recent studies highlighting its potential role in fostering EI-related competencies (Rico-González, 2023). These findings have important practical implications. Arab universities could integrate EI-based training programs into student development initiatives. Such interventions may not only promote academic success but also enhance emotional regulation and stress management, especially in highpressure or collectivist environments.

Our multi-group analysis confirmed both configural and metric invariance of the measurement models across the three national samples, indicating that Emotional Intelligence (EI), Psychological WellBeing (WB), and Psychological Distress (PD) were conceptualized and interpreted similarly across cultural contexts. This supports the validity of structural comparisons and underscores the importance of culturally grounded psychological models (Byrne, 2012; Cheung and Rensvold, 2002; Vandenberg and Lance, 2000). However, the lack of scalar invariance restricts the interpretability of cross-national mean-level comparisons. It is important to exercise caution when comparing average scores of psychological constructs across countries because this finding implies that cultural response styles may affect how people from various Arab contexts respond to self-report measures.

The mediation model received general support, nevertheless the strength of mediation varied across countries. Specifically, well-being mediated the relationship between emotional intelligence and psychological distress in Kuwait, compared to Oman or Jordan. This suggests that culture has an impacts on how emotional intelligence affects mental health, possibly due to cultural norms on emotional expression or differing stress management styles (Matsumoto, 2006; Mesquita and Walker, 2003). These differences show the need to adapt emotional intelligence interventions to specific cultures.

The findings of this study should be read with a few considerations in mind. Because the design was cross-sectional, it provides only a point-in-time view of the links between emotional intelligence, wellbeing, and psychological distress. This means we cannot draw conclusions about cause and effect. Future studies that follow students over time would help clarify the direction of these relationships and test whether the mediation model holds in the long term (Maxwell and Cole, 2007). Another point is that while the measures showed consistency across countries at some levels, scalar invariance was not achieved. This limits our ability to compare mean scores across cultures, so cross-country findings should be interpreted with caution. Future work could test partial scalar invariance, when theoretically sound, to strengthen such comparisons. The study also relied only on self-report questionnaires, which may be influenced by biases such as social desirability. This issue may be especially important in collectivist cultural contexts, where people sometimes adjust their emotional reporting to maintain social harmony (Harzing, 2006; Podsakoff et al., 2003). Using additional methods, such as peer or instructor ratings or behavioral indicators, could help provide a fuller and more reliable picture (Lindquist et al., 2016). Finally, the sample was limited to students from three Arab countries. While this adds important regional knowledge, it reduces the generalizability of the findings. Broader studies including more diverse Arab and non-Arab groups could enrich understanding of how cultural contexts shape emotional functioning and mental health (Matsumoto and Hwang, 2012). Overall, these considerations highlight areas for further research but do not lessen the contributions of the present study. By showing the role of well-being as a mediator and pointing to cultural differences, the findings provide both theoretical and practical insights. Most importantly, they highlighted the value of culturally sensitive strategies in higher education that build emotional skills as a way to support student mental health.

## Conclusion

This study found that emotional intelligence (EI) plays an important role in enhancing well-being and reducing stress, anxiety, and depression among physical education students in Oman, Kuwait, and Jordan. The results also suggest that cultural differences may shape how emotional intelligence is linked to well-being and psychological distress.

These findings show that emotional intelligence can help university students maintain better mental health, especially in cultures that focus on group harmony. Teaching students how to understand and manage their emotions could make them more resilient and better able to handle academic and personal challenges.

This research enhances existing knowledge by exploring emotional intelligence in the context of Arab university students, an area that has received much less attention compared to Western cultures.

### Limitations and future directions

While this study provides meaningful insights, it also has several limitations. First, its cross-sectional design limits our ability to determine the direction of the causal relationships. Future research should adopt longitudinal designs to better understand how these relationships evolve over time. Second, relying on self-report questionnaires may have introduced social desirability bias, particularly in collectivist cultures, such as Arab societies, where individuals may suppress emotional expression to maintain social harmony.

Moreover, the study sample was limited to students from only three Arab countries. Including participants from additional Arab and non-Arab countries in future research would enhance the generalizability of the findings and allow for richer cross-cultural comparisons.

Future studies should also explore how cultural norms shape emotional expression and examine how culturally tailored interventions can support psychological well-being in diverse educational settings.

## Data Availability

The raw data supporting the conclusions of this article will be made available by the authors, without undue reservation.
