# Correction: Structural relationships between emotional intelligence, well-being, and psychological distress: a multi-group SEM study among undergraduate physical education students in three Arab countries

**DOI:** 10.3389/fpsyg.2025.1722700

**Published:** 2025-10-24

**Authors:** Kashef Zayed, Ehab Omara, Mahfoodha Al Kitani, Ali Al-Yaaribi, Khalifa Al-Jadidi, Abdul Rahim Daroushi, Majid Al Busafi, Amin Gaafar, Heba El-Ashkar, Badriya Al-Hadabi, Ezzedin Ali

**Affiliations:** ^1^Department of Physical Education and Sport Sciences, College of Education, Sultan Qaboos University, Muscat, Oman; ^2^Department of Psychology, College of Education, Sultan Qaboos University, Muscat, Oman; ^3^Humanities Research Center, Sultan Qaboos University, Muscat, Oman

**Keywords:** emotional intelligence, psychological well-being, psychological distress, cross-cultural, structural equation modeling, university students

Authors Khalifa Al-Jadidi, Majid Al Busafi, Heba El-Ashkar and Ezzedin Ali were erroneously spelled as Khalifa Mubarak Al-Jadidi, Majid Said Al Busafi, Heba Al-Ashkar and Ezzedin Mohamed Ali.

The original version of this article has been updated.

